# Hirudo Lyophilized Powder Ameliorates Renal Injury in Diabetic Rats by Suppressing Oxidative Stress and Inflammation

**DOI:** 10.1155/2021/6657673

**Published:** 2021-02-22

**Authors:** Fan Yang, Yachun Li, Shuai Guo, Yongmei Pan, Cuihuan Yan, Zhiqiang Chen

**Affiliations:** ^1^Hebei University of Chinese Medicine, Shijiazhuang, Hebei 050091, China; ^2^Affiliated Hospital of Hebei University of Traditional Chinese Medicine, Shijiazhuang, Hebei 050011, China

## Abstract

As diabetic nephropathy (DN) is one of the most common and destructive microvascular complications of diabetes mellitus, the goal of this study, therefore, was to investigate the renal protective effect and latent mechanisms of Hirudo lyophilized powder on diabetic rats. In this study, all rats were randomly assigned into the control group and diabetic group. The rats of diabetic group were injected with low-dose STZ (35 mg/kg) intraperitoneal plus high-fat diet to induce diabetes. Then, the successful diabetic model rats were weighed and randomly assigned into four groups: (1) diabetic model group (DM group); (2) Hirudo lyophilized powder 0.3 g/kg treatment group (SL group); (3) Hirudo lyophilized powder 0.6 g/kg treatment group (SM group); (4) Hirudo lyophilized powder 1.2 g/kg treatment group (SH group). Their fasting blood glucoses (FBG) were measured every 4 weeks. After treatment with Hirudo lyophilized powder at a corresponding dose once a day for 16 weeks, their metabolic and biochemical as well as oxidative stress parameters were tested, and the kidney weight (KW)/body weight (BW) was calculated. The renal tissues were used for histological, mRNA, and protein expression analysis. The results showed that Hirudo lyophilized powder could protect against the structural damages and functional changes of diabetic renal tissue by inhibiting oxidative stress, inflammation, and fibrosis. Furthermore, it was found in the further research that inhibiting the NOX4 expression and JAK2/STAT1/STAT3 pathway activation might be the underlying mechanisms. Collectively, Hirudo lyophilized powder might be a promising therapeutic agent for the treatment of DN.

## 1. Introduction

Diabetes mellitus is an epidemic disease worldwide. The International Diabetes Federation reported that there were approximately 451 million diabetic patients worldwide in 2017, and the figure was expected to increase to 693 million by 2045 [1]. Persistent hyperglycemia and long-term metabolic disorders will cause damage to multiple organs and tissues [2]. Among them, diabetic nephropathy (DN), which seriously endangers human health, is one of the most common and destructive microvascular complications, and it is also the primary cause of end-stage renal disease [3]. Approximately 20–40% of patients develop DN after the onset of diabetes [4]. Currently, strict glycemic and blood pressure control constitute the mainstay of management for DN [3]. However, DN still occurs in a significant portion of diabetic patients. Therefore, it may be useful and necessary to study therapies specifically target for DN.

As we all know, oxidative stress has been submitted to be one of the key roles in the development and progression of DN [5]. Oxidative stress is caused by the increase of ROS and the deficiency of antioxidant defense system, which results in unwanted modifications to lipids, proteins, DNA, etc. [5]. Multiple studies have shown that NOX4, a subtype of nicotinamide adenine dinucleotide phosphate oxidase (NADPH oxidase, NOx), was the main source of ROS in renal cells [6]. In a rodent model of DN, the global genetic ablation of NOX4 or pharmacological suppression of its activity could significantly attenuate proteinuria, glomerulosclerosis, and extracellular matrix (ECM) protein accumulation by reducing the production of ROS [6].

Moreover, oxidative stress and inflammation are inextricably linked, which can cause inflammation through a variety of mechanisms [7]. It was reported that the intracellular ROS generation derived from NADPH oxidases could regulate the Janus kinase-signal transducers and activators of transcription (JAK/STAT) pathway [8]. The JAK/STAT pathway is a hub for regulating the expressions of inflammatory genes, which can initiate the transcription of various inflammatory genes [9]. Currently, there are four members of JAK kinases (JAK1-3 and TYK2) and seven STATs (STAT1-4, 5a, 5b, and 6) recognized in mammals [9]. Among them, the JAK2/STAT1/STAT3-dependent axis is the most explicitly recognized form during the occurrence and progression of DN [10, 11].

Accumulating experiment and clinical studies have demonstrated that many traditional Chinese medicines (TCM) or active ingredients of TCM had preventive and therapeutic effects on kidney damage caused by diabetes [12]. Blood stasis blocking the collaterals of kidney is one of the main pathogeneses in DN and has been widely approved by the academic circles of TCM [13]. Our previous research also confirmed that Huayu Tongluo herbs had protective effect on renal tissues of diabetic rats [13]. Hirudo, also known as Chinese medicinal leech, is a commonly used animal-derived TCM for promoting blood circulation and removing blood stasis [14]. It is widely distributed in rivers, lakes, and other wetland environments. Hirudo was first recorded in Shen Nong's Herbal Classic, which can be traced back to the Eastern Han Dynasty, and it had a history of medical application in China for thousands of years. In the history of European medicine, Hirudo-therapy was once thought to be effective for all diseases [15]. Modern research has demonstrated that Hirudo contained many pharmacological effects such as anticoagulation, antiplatelet aggregation, antithrombotic, anti-inflammatory and analgesia, antitumor as well as hemorheology improvement [16]. Dozens of active ingredients, such as hirudin, pteridine, phosphatidylcholine, sphingolipids, and sterols, have been identified from Hirudo [16]. However, the current basic research of Hirudo was mainly its protective effect on arterial blood vessels [17]. There were scarce data on the beneficial effects of Hirudo in DN, so the goal of this study was to investigate the protective effect of Hirudo on the kidneys in diabetic rats. In addition, we also studied the effects of Hirudo on NOX4 and JAK/STAT pathway to elucidate the possible mechanism.

## 2. Materials and Methods

### 2.1. Animals

A total of 48 (4–5 weeks, SPF grade) male Sprague-Dawley (SD) rats weighing 70 ± 10 g were provided by Beijing Vital River Laboratory Animal Technology Co., Ltd. (Beijing, China, license number: SCXK (Jing) 2016–0006). All SD rats were bred in a light-controlled (12 h:12 h light/dark alter) room with temperature 24 ± 1°C and humidity 50%–70%. The rats were allowed free access to standard food and water. This study was performed in accordance with the China Animal Welfare Legislation and approved by the Ethics Committee of Hebei University of Chinese Medicine (number DWLL2015002), China. Every effort was made to minimize suffering.

### 2.2. Experimental Drug

The Hirudo lyophilized powder in this study was all derived from blood-sucking Hirudo nipponica Whitman and provided by Chongqing Duo Putai Pharmaceutical Co., Ltd. (approval certificate: Z10970056).

### 2.3. Animal Modeling and Grouping

**Figure 1:**
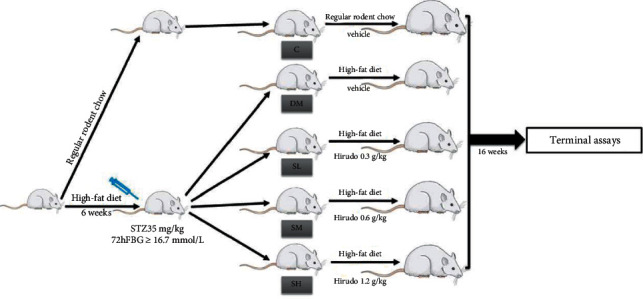
Animal experimental design.

The animal experimental scheme was depicted in Figure 1. After one week of adaptive rearing with regular rodent chow (protein 21.1%, fat 4.5%, carbohydrate 60.6%; Botai Hongda Biotechnology Co., Ltd., Beijing, China, http://biotech-hd.cn.gongchang.com), all rats were randomly assigned into the control group (C group, *n* = 8) and diabetic group (*n* = 40) according to the random number table method. The rats in the C group were still bred with regular rodent chow, while the diabetic group rats were bred with high-fat diet (HFD, protein 24.2%, fat 25.4%, carbohydrate 42.1%; Botai Hongda Biotechnology Co., Ltd., Beijing, China, http://biotech-hd.cn.gongchang.com) for the initial period of 6 weeks according to the method designed by us previously [13]. After fasting overnight, the diabetic group rats were administered intraperitoneal injection of low-dose streptozotocin (STZ, Enzo Life Science Co., Ltd., USA, 35 mg/kg) diluted with 0.1 M cold citrate buffer (pH 4.3). Meanwhile, the rats in the C group were injected with the corresponding volume of citrate buffer. Their blood glucose concentration was measured 72 h later. When the rat's concentration of blood glucose was above 16.7 mmol/L, it was identified as a successful diabetic rat model and the rat was selected for the following study. Two rats died within 72 hours after STZ injection, while another was excluded due to unsuccessful modeling. The successful diabetic model rats were weighed and randomly assigned into four groups: (1) diabetic model group (DM group, *n* = 10); (2) Hirudo lyophilized powder 0.3 g/kg treatment group (SL group, *n* = 9); (3) Hirudo lyophilized powder 0.6 g/kg treatment group (SM group, *n* = 9); (4) Hirudo lyophilized powder 1.2 g/kg treatment group (SH group, *n* = 9). The dose of Hirudo lyophilized powder administered in rats was chosen based on previous studies [18, 19]. Hirudo lyophilized powder gavage was administered once a day at a corresponding dose for 16 weeks while the C and DM group rats were given an equal volume of drinking water. In addition, the rats in the C group continued to be fed with regular rodent chow while the other diabetic rats were bred with HFD until the end of this experiment. During the experiment, no other intervention was given.

### 2.4. Specimen Collection

After 16 weeks of medication, the urine of rats was collected in a metabolic cage. Then, the rats were anesthetized with 3% isoflurane inhalation after fasting overnight. Blood samples were collected from the femoral artery, which resulted in rats' death because of bleeding shock, while rats that did not die were sacrificed by cervical dislocation under anesthesia. The serum was separated from blood samples and then stored at −20°C for subsequent biochemistry. Kidneys were quickly separated, weighed, and washed with cold saline. Then, the kidney was divided into several parts, one part was fixed in 4% paraformaldehyde for histological analysis and the others were stored in liquid nitrogen for oxidative stress analysis and protein and mRNA extraction. Urine, blood, and renal tissue samples were analyzed and measured by blind method.

### 2.5. Determination of Metabolic and Biochemical Parameters

The kidney weight (KW)/body weight (BW) was calculated after all rats were sacrificed. The rats' FBG levels were estimated at 4th, 8th, 12^th^, and 16th weeks with rapid blood glucose meter (Johnson & Johnson Medical Ltd., USA). The levels of creatinine (SCr), urea nitrogen (BUN), cholesterol (CHO), and triglyceride (TG) were determined by a Hitachi automatic biochemical analyzer (7600–020, Hitachi, Tokyo, Japan). A urine protein test kit (Nanjing Jiancheng Bioengineering Institute, Jiangsu, China) was used to measure urinary protein quantity, and the urinary protein quantity was expressed as milligrams per 24 h.

### 2.6. Assessment of Renal Oxidative and Antioxidant Parameters

Some renal tissues taken out from liquid nitrogen were homogenized in ice-cold normal saline, and the supernatant was collected by centrifuging (4000 g, 10 min, 4°C) the homogenate. The content of malondialdehyde (MDA) and the activities of superoxide dismutase (SOD) and glutathione peroxidase (GSH-PX) were analyzed by related detection kits (Nanjing Jiancheng Bioengineering Institute, Jiangsu, China) based on the manufacturer's instructions.

### 2.7. Western Blot Assay

Total protein was prepared from the renal cortex of rats by ice-cold RIPA lysis buffer (Beyotime Institute of Biotechnology, Shanghai, China) and HALT protease/phosphatase inhibitor cocktail (Servicebio, Wuhan, China). Then, the total protein concentration was quantified by the BCA assay (Tiangen Biotech, Beijing, China). Afterwards, the protein sample was diluted with 5× loading buffer and denatured at 100°C for 5 minutes. Equal quantity of protein was fractionated by 10% sodium dodecyl sulphate-polyacrylamide gel electrophoresis (SDS-PAGE) and then transferred onto the polyvinylidene difluoride (PVDF) membranes (Millipore Corporation, Bedford, MA, USA). After blocked with 5% nonfat milk for two hours and washed with TBST for three times, the membranes were incubated in primary antibodies overnight at 4°C and then in HRP-labeled goat anti-rabbit IgG secondary antibodies (ZSGB-BIO, Beijing, China) for 1 hour at room temperature, respectively. The protein bands were visualized by the ECL kit (Millipore, Bedford, MA) and captured by the ImageQuant LAS4000 imaging system (GE, USA). The *β*-actin antibody was used as a loading control, and relative density of each band was performed and quantified by the ImageJ software (NIH, Bethesda, MD, USA). The primary antibodies used are as follows: JAK2 (3230, Cell Signaling Technology, USA, 1 : 500), p-JAK2 (3776, Cell Signaling Technology, USA, 1 : 500), STAT1 (14994, Cell Signaling Technology, USA, 1 : 500), p-STAT1 (7649, Cell Signaling Technology, USA, 1 : 500), STAT3 (4904, Cell Signaling Technology, USA, 1 : 500), p-STAT3 (9145, Cell Signaling Technology, USA, 1 : 500), NOX4 (ab133303, Abcam, UK, 1 : 1000), collagen IV (ab6586, Abcam, UK, 1 : 1000), FN (A12932, ABclonal, Wuhan, China, 1 : 800), TGF-*β*1 (ab215715, Abcam, UK, 1 : 1000), and *β*-actin antibody (AF0003, Beyotime, Shanghai, China, 1 : 1000).

### 2.8. Quantitative Real-Time Polymerase Chain Reaction (RT-PCR) Assay

Total RNA of the rat renal cortex was extracted by the TRIzol reagent (Servicebio, Wuhan, China). Its concentrations were analyzed by a Nanodrop 2000C spectrophotometer (Thermo Scientific, Wilmington, USA). Then, the RNA was reverse-transcribed into cDNA by a Servicebio^®^RT First Strand cDNA Synthesis Kit (Servicebio, Wuhan, China) according to the manufacturer's instructions. qRT-PCR was operated with the 2×SYBR Green qPCR Master Mix (High ROX) (Servicebio, Wuhan, China) by an ABI Prism 7300 Sequence Detection System (Applied Biosystems, USA). Gene-specific primers (Table 1) were designed and synthesized by Servicebio Technology Co., Ltd. (Wuhan, China). Each reaction was implemented in triplicate, and the relative levels of target genes were calculated by the 2^−ΔΔCt^ method.

### 2.9. Immunohistochemistry and Immunofluorescence

The renal tissue sections were dewaxed with xylene, dehydrated with different concentrations of alcohol, and then rinsed with distilled water. The epitopes were retrieved by microwave and the sections were incubated with 3% hydrogen peroxide deionized water to inhibit endogenous peroxidase activity. Then, the sections were blocked with goat serum at 37°C for 30 min and incubated with primary antibodies against collagen III (1 : 250, rabbit, GB111629, Servicebio, Wuhan, China) and fibronectin (1 : 100, rabbit, A12932, ABclonal, Wuhan, China) at 4°C overnight. After washing, the sections were incubated with HRP-conjugated goat anti-rabbit IgG secondary antibody (1 : 200, GB23303, Servicebio, Wuhan, China) at 37°C for 30 min. The sections were rinsed again with PBS and visualized with diaminobenzidine (DAB). Hematoxylin was used as a counterstaining. Finally, the stained sections were observed under a microscope in a blind manner.

For immunofluorescence analysis, renal tissues were fixed in 4% paraformaldehyde overnight and then embedded in paraffin and sectioned. After dewaxing and dehydration, tissue sections were incubated with primary antibodies against inducible nitric oxide synthase (iNOS, 1 : 100, rabbit, bs-2072R, Bioss, Beijing, China) and CD68 (1 : 100, rabbit, bs-0649R, Bioss, Beijing, China) at 4°C overnight. Then, the sections were rinsed in PBS and incubated with Alexa Flour®594-conjugated goat anti-rabbit IgG secondary antibody (1 : 50, ZF-0516, ZSGB-BIO, Beijing, China) at 37°C for 1h. DAPI (Boster, Wuhan, China) was used as a counterstaining. Finally, the sections were observed with an Olympus IX71 fluorescence microscope (Olympus, Japan). Immunohistochemistry and immunofluorescence staining were analyzed quantitatively by Image-Pro Plus 6.0 software (Media Cybernetics, USA).

### 2.10. Renal Histopathology Analysis

The renal tissue was dehydrated and embedded in paraffin after fixed in 4% paraformaldehyde for 24 hours. The embedded tissue was cut into 4 *μ*m-thick sections and stained with hematoxylin-eosin (HE) and Masson's trichrome, respectively. The procedure of section staining was carried out according to the instructions. The morphology of renal tissues was observed by an optical microscope (Olympus Co., Ltd., Tokyo, Japan) in a blind manner. The Image-Pro Plus 6.0 software was used to analyze the pathological images of renal tissues stained by Masson's trichrome.

### 2.11. Statistical Analysis

All data were analyzed by SPSS software (version 26.0, IBM, Chicago, IL, USA) and expressed as means ± standard deviation (SD). GraphPad Prism software (GraphPad Software Inc.,V5.02, San Diego, CA, USA) was utilized for graphing. The results were analyzed by one-way ANOVA. The LSD tested for the differences between two groups and the Levene method was used for homogeneity test of variance. A value of *P* < 0.05 was defined as statistically significant for all analyses.

## 3. Results

### 3.1. Effects of Hirudo Lyophilized Powder on Metabolic and Biochemical Parameters of Diabetic Rats

**Figure 2:**
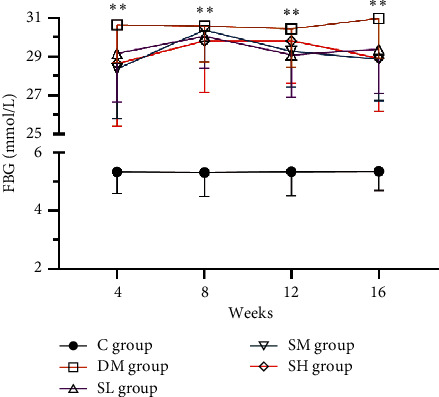
FBG changes of tendency in different groups. The changes of tendency in rats' FBG every 4 weeks. Data are presented as means ± SD (*n* = 7 in DM and SL groups, *n* = 8 in the other groups). ^*∗∗*^*P* < 0.01 vs. C group.

STZ injection combined with HFD in rats can simulate the characteristics of human type 2 diabetes and its complications, such as proteinuria, impaired renal function, glomerular hypertrophy, and glomerulosclerosis [20]. After intervention with Hirudo lyophilized powder for 16 weeks, the metabolic and biochemical parameters of rats are shown in Table 2. Compared with the C group rats, the ratio of KW/BW, blood lipids (CHO, TG), renal function (BUN, SCr), and 24 h-UTP were all significantly increased in DM group rats (*P* < 0.01). However, compared with the DM group rats, Hirudo lyophilized powder treatment attenuated the renal injury parameters (KW/BW, BUN, SCr, and 24 h-UTP) in a dose-dependent manner (*P* < 0.05, *P* < 0.01). Furthermore, we did not find any changes of CHO in the SL group rats when compared with the DM group rats (*P*>0.05), while the CHO and TG levels in SM and SH groups rats as well as the TG levels in SL group rats displayed a significant reduction (*P* < 0.01). Finally, as displayed in Figure 2, we estimated rats' FBG at 4th, 8th, 12^th^, and 16th weeks, respectively. The FBG was normal in C group rats, while it was obviously increased in DM group rats and Hirudo lyophilized powder treatment group rats (*P* < 0.01), which indicated administration of Hirudo lyophilized powder had no effect on FBG (*P* > 0.05). Collectively, our data showed that Hirudo lyophilized powder could improve the renal injury parameters and blood lipids, but it had no effect on the blood glucose concentration.

### 3.2. Effects of Hirudo Lyophilized Powder on Renal Histopathology

**Figure 3:**
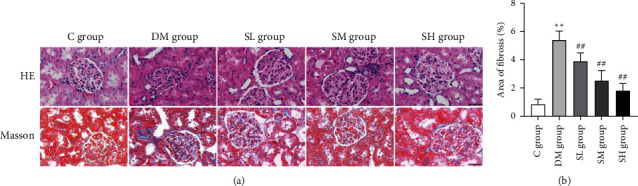
Effects of Hirudo lyophilized powder on renal histopathology of diabetic rats. The SL, SM, and SH group rats were dosed by oral gavage once daily for 16 weeks with Hirudo lyophilized powder. The C and DM group rats were administered the same volume of vehicle used to prepare the test medications. (a) Pathological morphology of renal tissues in each group rats (×400); scale bar, 50 m. (b) The fibrosis area of renal tissues in each group rats. Data are presented as means ± SD; ^*∗∗*^*P* < 0.01 vs. C group; #*P* < 0.05 and ##*P* < 0.01 vs. DM group.

In addition to the results of renal injury parameters, we further evaluated the histopathological changes of rat kidneys. As shown in Figure 3(a), the C group rats showed no obvious histopathological changes in the glomerulus, renal tubules, or interstitium. In contrast, the DM group rats showed the mesangial expansion with glomerular hypertrophy, basement membrane thickening, tubular atrophy/dilation, and infiltration of inflammatory cells. Notably, with the administration of Hirudo lyophilized powder, the histopathological changes were weakened to varying degrees in each treatment group rats. Furthermore, Masson staining indicated the deposition of collagen in the renal tissues, which was used to evaluate the development of fibrosis. As shown in Figure 3(b), low level of collagen was found in C group rats. However, the collagen accumulation was found more extensive in the renal tissues of DM group rats (*P* < 0.01). In contrast, Hirudo lyophilized powder treatment was associated with only slight collagen deposition (*P* < 0.01). These results suggested that renal histological abnormalities could be significantly ameliorated by Hirudo lyophilized powder treatment in diabetic rats, and this amelioration is independent of hyperglycemia effect.

### 3.3. Effects of Hirudo Lyophilized Powder on Oxidative Stress and NOX4 of Diabetic Rats

**Figure 4:**
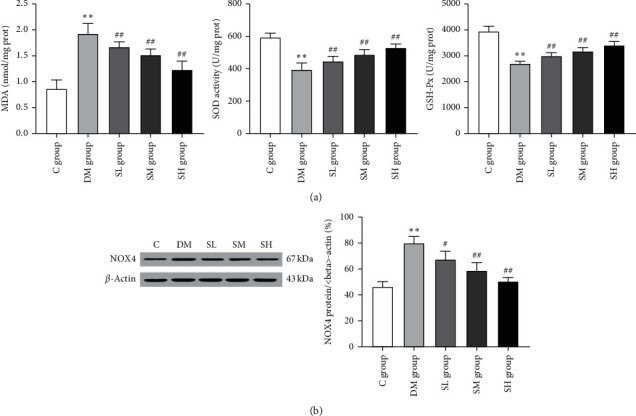
Effects of Hirudo lyophilized powder on oxidative stress parameters and NOX4 protein in rat renal tissues. The SL, SM, and SH group rats were dosed by oral gavage once daily for 16 weeks with Hirudo lyophilized powder. The C and DM group rats were administered the same volume of vehicle used to prepare the test medications. (a) The levels of MDA and the activities of SOD and GSH-Px in each group rats. Data are presented as means ± SD (*n* = 7 in DM and SL groups, *n* = 8 in the other groups). (b) The protein expression and quantitative analysis of NOX4 in each group rats. Data are presented as means ± SD (*n* = 3). ^*∗∗*^*P* < 0.01 vs. C group; #*P* < 0.05 and ##*P* < 0.01 vs. DM group.

Oxidative stress has been thought to be one of the major mechanisms underlying the pathogenesis of DN [5]. To identify the influence of Hirudo lyophilized powder on oxidative stress in DN, we measured the levels of oxidative index MDA and the activities of antioxidant enzymes GSH-PX, SOD. As shown in Figure 4(a), compared with the C group rats, the levels of lipid peroxidation product (MDA) were significantly upregulated in the renal tissues of DM group rats (*P* < 0.01). Meanwhile, the activities of antioxidant enzymes (GSH-PX, SOD) were also significantly reduced (*P* < 0.01). However, the administration of Hirudo lyophilized powder significantly reduced oxidative index (MDA) levels and recovered the activities of antioxidant enzymes (GSH-PX, SOD) in a dose-dependent manner (*P* < 0.01).

NOX4 is believed to be the major enzyme leading to renal oxidative stress [6]. Therefore, we tested the expression of NOX4 by western blotting. As shown in Figure 4(b), compared with the C group rats, the expression of NOX4 was significantly upregulated in DM group rats (*P* < 0.01), while the administration of Hirudo lyophilized powder significantly reduced the expression of NOX4 in each treatment group rats (*P* < 0.05, *P* < 0.01). Collectively, our data suggested that Hirudo lyophilized powder might reduce oxidative stress by partially inhibiting the expression of NOX4.

### 3.4. Effects of Hirudo Lyophilized Powder on the Inflammatory Cytokine Levels and Macrophage Infiltration of Diabetic Rats

**Figure 5:**
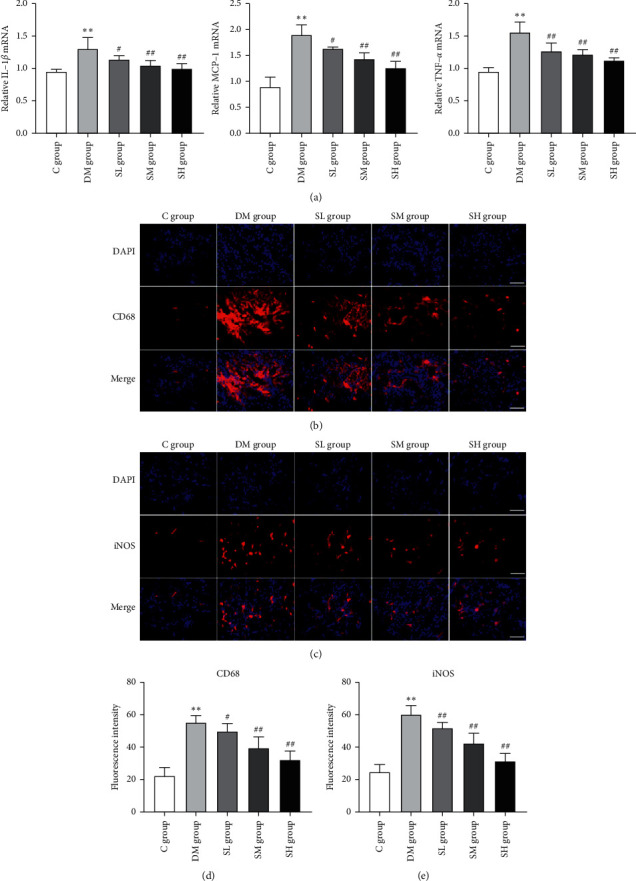
Effects of Hirudo lyophilized powder on the inflammatory cytokine levels and macrophage infiltration in rat renal tissues. The SL, SM, and SH group rats were dosed by oral gavage once daily for 16 weeks with Hirudo lyophilized powder. The C and DM group rats were administered the same volume of vehicle used to prepare the test medications. (a) The mRNA levels of IL-1*β*, MCP-1, and TNF-*α* in each group rats. Data are presented as means ± SD (*n* = 3). (b, c) Immunofluorescence staining for CD68 and iNOS in the renal tissues of each group rats; scale bar, 100 m. (d, e) The fluorescence intensity of CD68 and iNOS in each group rats. Data are presented as means ± SD. ^*∗∗*^*P* < 0.01 vs. C group; #*P* < 0.05 and ##*P* < 0.01 vs. DM group.

The production of inflammatory cytokines is usually caused by the increased expressions of related inflammatory genes. Therefore, we specifically studied their levels in rat renal tissues by qRT-PCR. As shown in Figure 5(a), compared with the C group rats, the mRNA levels of IL-1*β*, MCP-1, and TNF-*α* were increased significantly in DM group rats (*P* < 0.01), while compared with the DM group rats, all the mRNA levels of IL-1*β*, MCP-1, and TNF-*α* were effectively reversed during intervention with Hirudo lyophilized powder (*P* < 0.05, *P* < 0.01).

In addition, we investigated the levels of macrophage infiltration in rat renal tissues by immunofluorescence. Macrophages are one of the important inflammatory cell types. In this study, CD68, as a macrophage surface marker, was used to observe macrophage infiltration in the rat renal tissues. As shown in Figures 5(b) and 5(d), no significant infiltration of CD68-positive macrophage was detected in the renal tissues of C group rats. In contrast, abnormal macrophage infiltration was observed in DM group rats (*P* < 0.01), while Hirudo lyophilized powder treatment significantly reduced the abnormal macrophage infiltration in the renal tissues of diabetic rats (*P* < 0.05, *P* < 0.01). Furthermore, iNOS is a cell-specific marker of M1 type macrophage activation, which can promote secretion of the inflammatory factors [21]. As shown in Figures 5(c) and 5(e), compared with the C group rats, the fluorescence intensity of iNOS significantly increased in DM group rats (*P* < 0.01), but Hirudo lyophilized powder treatment significantly inhibited this effect (*P* < 0.01).

### 3.5. Effects of Hirudo Lyophilized Powder on the Renal Fibrosis-Related Proteins of Diabetic Rats

**Figure 6:**
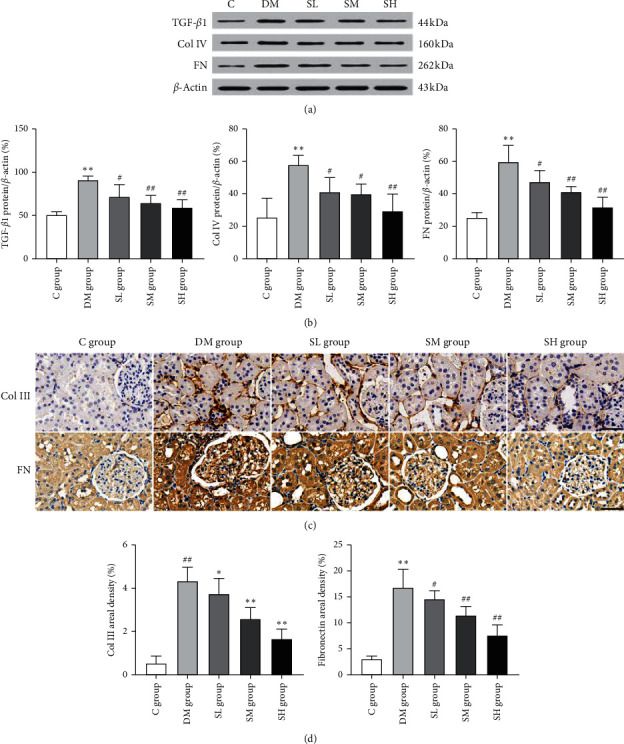
Effects of Hirudo lyophilized powder on the TGF-*β*1 and ECM proteins in rat renal tissues. The SL, SM, and SH group rats were dosed by oral gavage once daily for 16 weeks with Hirudo lyophilized powder. The C and DM group rats were administered the same volume of vehicle used to prepare the test medications. (a, b) The protein expressions and quantitative analysis of TGF-*β*1, collagen IV, and FN in each group rats. Data are presented as means ± SD (*n* = 3). (c, d) The immunohistochemistry staining and quantitative analysis of collagen III and FN in the renal tissues of each group rats; scale bar, 50 m. Data are presented as means ± SD. ^*∗∗*^*P* < 0.01 vs. C group; #*P* < 0.05 and ##*P* < 0.01 vs. DM group.

As we all know, TGF-*β*1 is the most effective fibrotic growth factor. The collagen protein and fibronectin (FN) are important components of ECM, both are closely related to renal fibrosis. As shown in Figures 6(a) and 6(b), the expressions of TGF-*β*1, collagen IV, and FN were significantly upregulated in the DM group rats when compared with the C group rats (*P* < 0.01), while compared with the DM group rats, the expressions of TGF-*β*1, collagen IV, and FN were effectively reversed after intervention with Hirudo lyophilized powder (*P* < 0.05, *P* < 0.01). Consistent with this result, as shown in Figures 6(c) and 6(d), immunohistochemistry staining also demonstrated that the collagen III and FN deposition were significantly reduced in renal tissues of each treatment group rats after intervention with Hirudo lyophilized powder (*P* < 0.05, *P* < 0.01). Therefore, it could be concluded that Hirudo lyophilized powder attenuated renal fibrosis by inhibiting the TGF-*β*1 expression and ECM deposition.

### 3.6. Effects of Hirudo Lyophilized Powder on the JAK2/STATs Pathway of Diabetic Rats

**Figure 7:**
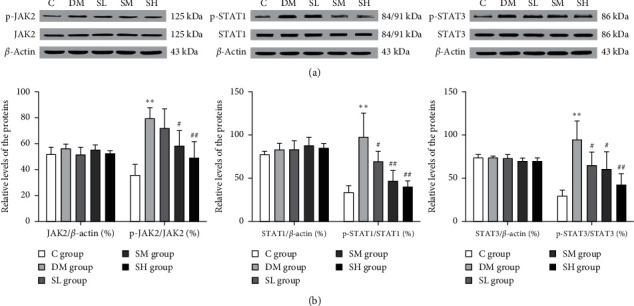
Effects of Hirudo lyophilized powder on the JAK2/STATs pathway in rat renal tissues. The SL, SM, and SH group rats were dosed by oral gavage once daily for 16 weeks with Hirudo lyophilized powder. The C and DM group rats were administered the same volume of vehicle used to prepare the test medications. (a) The protein expressions of JAK2/STAT1/STAT3 pathway in each group rats. (b) The quantitative analysis of JAK2/STAT1/STAT3 pathway in each group rats. Data are presented as means ± SD (*n* = 3). ^*∗∗*^*P* < 0.01 vs. C group; #*P* < 0.05 and ##*P* < 0.01 vs. DM group.

As mentioned above, Hirudo lyophilized powder showed anti-inflammatory and antifibrosis effects. Therefore, we detected the JAK/STAT pathway associated with inflammation and fibrosis by western blotting. As shown in Figure 7, western blotting results indicated that the expressions of p-JAK2, p-STAT1, and p-STAT3 were significantly increased in DM group rats when compared with the C group rats (*P* < 0.01), which indicated the activation of JAK2/STAT1/STAT3 pathway. Of note, Hirudo lyophilized powder significantly decreased the expressions of p-JAK2, p-STAT1, and p-STAT3 (*P* < 0.05, *P* < 0.01), whereas the levels of JAK2, STAT1, and STAT3 had no difference in each group rats (*P* > 0.05). Collectively, we concluded that Hirudo lyophilized powder might inhibit the overactivation of JAK2/STATs pathway.

## 4. Discussion

DN is the most common microvascular complication of diabetes mellitus [3]. It can cause mesangial matrix expansion, glomerulosclerosis, renal interstitial inflammation and fibrosis, and eventually lead to renal failure. Clinically, proteinuria is the main clinical manifestation of DN. The KW/BW ratio is used as an indicator of the renal hypertrophy index [22]. BUN and SCr are also the most widely used indicators of renal function. The increases of these parameters are considered as a marker of the renal injury in DN. In our study, low-dose intraperitoneal injection of STZ plus HFD treatment resulted in significant hyperglycemia, increasing the renal hypertrophy index, impairment of renal function (BUN, SCr), and elevation of urine protein excretion, as well as abnormal renal tissue structure in diabetic rats. These data demonstrated that the DN model rat was successfully established. Of note, the renal hypertrophy index decreased, renal function improved, urine protein excretion decreased significantly, and related pathology ameliorated after administration with Hirudo lyophilized powder. However, Hirudo lyophilized powder did not affect blood glucose levels in diabetic rats. In addition, abnormal lipid levels contribute to the advancement of DN by increasing renal lipotoxicity [23]. After treatment with Hirudo lyophilized powder, the blood lipid levels of diabetic rats were significantly reduced.

Oxidative stress plays an important role in the pathogenesis of DN [5]. Under physiological conditions, pro-oxidants and antioxidants interact to maintain equilibrium. A slight of ROS can be eliminated quickly by antioxidant substances (such as superoxide dismutase, catalase, and glutathione peroxidase) in the tissues, while in diabetes mellitus, the increases of pro-oxidants and the reduction of antioxidants lead to an excessive production in ROS. Subsequently, the basic life substances such as lipids, nucleic acids, and proteins are oxidized, which brings about changes in their structure and function. This is the ROS-induced oxidative stress. Accumulating evidences have indicated that NOX4 was the main source of ROS in renal cells [6]. In a high glucose environment, silencing or inhibiting NOX4 in human podocytes could significantly reduce the production of ROS and the expressions of proinflammatory genes [24]. In our study, we observed that the expression of NOX4 protein was increased in diabetic rats, which was accompanied by the impairment of antioxidant enzymes (GSH-PX, SOD) activities and the upregulation of MDA content. However, Hirudo lyophilized powder at three different dosages significantly decreased NOX4 expression, enhanced antioxidant capacity, and decreased MDA content in diabetic rats, which indicated that Hirudo lyophilized powder was capable of eliminating oxidative stress by inhibiting the NOX4 enzyme.

Oxidative stress is inextricably linked to inflammation [7]. Endogenous ROS acts as a second messenger to stimulate the secretion of inflammatory cytokines [25]. Increasing evidences suggested that persistent chronic low-grade inflammation played a crucial role in the pathogenesis of DN [26]. Many kidney protective medicines were implicated in anti-DN activities by suppressing inflammatory responses [27]. The expressions of proinflammatory mediators such as TNF-*α*, IL-1*β,* and MCP-1 were upregulated in DN [28]. IL-1*β*, as an autocrine growth factor, has been demonstrated to be involved in inducing the proliferation of mesangial cells, which leads to glomerulosclerosis [29]. TNF-*α* facilitates the recruitment of monocytes and macrophages, irritates the production of ROS, and alters the permeability of endothelial cells, thereby bringing about the leakage of albumin [30]. MCP-1, as the most effective chemokine and macrophage activator, is diffusely considered to play a leading role in mediating the migration of monocytes and macrophages into renal tissues, which may result in immediate injury to renal tissues [31]. Furthermore, macrophages are one of the important inflammatory cell types. In DN, the main manifestation is aggregation of M1 proinflammatory macrophages in kidney tissues [32]. Clinical and experimental evidences have confirmed that the extent of progressive renal injury and fibrosis in DN was positively correlated with the number of renal macrophages infiltration [33]. The M1 macrophages further promote the secretion of proinflammatory factors such as MCP-1, TNF-*α,* and IL-1*β*, thus forming a positive feedback loop, as each begets and amplifies the other [34]. In our study, we observed significantly elevated proinflammatory cytokines IL-1*β*, TNF-*α,* and MCP-1, as well as massive CD68-positive and M1-type macrophage infiltration in the renal tissues of diabetic rats, while Hirudo lyophilized powder administration significantly reversed this effect. Consequently, our results demonstrated the potential anti-inflammatory function of Hirudo lyophilized powder in DN.

As mentioned above, the JAK/STAT pathway is a hub for regulating the expressions of inflammatory genes [9]. In addition, the JAK/STAT pathway has been shown to promote polarization of M1 macrophages [35]. Previous studies have demonstrated that the intracellular ROS generation derived from NADPH oxidase could regulate the JAK/STAT pathway [8]. JAK2 is a central moderator of oxidative stress-induced cell injury [36]. Multiple stimuli, including AGEs, cytokines, and Ang II, can activate the JAK2/STATs pathway through ROS to facilitate the progression of DN [37], while NOX and ROS inhibitors can significantly prevent the activation of JAK/STAT pathway and the expressions of some inflammatory genes [38]. In the present JAK/STAT pathway pattern, it is mainly composed of tyrosine kinase-related receptors, tyrosine kinase JAKs, and signal transducers and activators of transcription (STATs). When the ligands bind to a tyrosine kinase-associated receptor, JAKs become activated and then in turn phosphorylate the intracellular domain of the receptor and permit the recruitment and phosphorylation of STATs proteins. Phosphorylated STATs form dimers and translocate to the nucleus, which then regulate the expressions of target genes encoding inflammatory factors, chemokines, and adherence molecules [39]. JAK2 inhibition and STAT3 gene knockdown can significantly reduce the expressions of inflammatory factors and inflammatory cells [39, 40]. Our study demonstrated that the expressions of p-JAK2, p-STAT1, and p-STAT3 were significantly upregulated in the renal tissues of diabetic rats, which decreased a lot after intervention with Hirudo lyophilized powder at three different dosages. In summary, we reported for the first time that Hirudo lyophilized powder could inhibit JAK2/STAT1/STAT3 activation in the kidney of diabetic rats.

Glomerulosclerosis and interstitial fibrosis are the final outcome of DN, which is characterized by the excessive deposition of ECM, including FN and collagen [41]. The buildup of these ECM proteins eventually turns epithelial cells into fibroblasts in diabetic renal tissues, which leads to renal fibrosis [42]. Activation of JAK/STAT pathway can also enhance the production of TGF-*β*1, type IV collagen, and FN in mesangial cells [43]. It has been confirmed that two DNA binding sites of STAT3 were located in the promoter region of TGF-*β*1 gene and directly regulated the expression of TGF-*β*1 [44]. TGF-*β*1 is considered to be the most effective fibrotic molecule, which mediates glomerulosclerosis and tubulointerstitial fibrosis in DN by enhancing ECM synthesis [45]. Our results showed that Hirudo lyophilized powder could inhibit the expression of TGF-*β*1 in the renal tissues of diabetic rats. Meanwhile, the expressions of type III and IV collagen and FN were also reduced. Taken together, Hirudo lyophilized powder could effectively reduce TGF-*β*1 production and ECM accumulation in diabetic rats.

## 5. Conclusion

In summary, our study provided novel evidence that Hirudo lyophilized powder could protect against the structural damages and functional changes of diabetic renal tissues by inhibiting oxidative stress, inflammation, and fibrosis. Further research found that the underlying mechanisms might be related to inhibiting the NOX4 expression and JAK2/STAT1/STAT3 pathway activation. Thus, these findings provided a potential target of Hirudo lyophilized powder for prevention or treatment of DN. But our research still had some limitations. For example, we only focused on the signal pathways of NOX4 and JAK/STAT, and the corresponding signal pathway inhibitors were also not selected. Therefore, more investigations should be conducted to make the experiment more precise and meaningful.

## Figures and Tables

**Table 1:** Primers for rat genes used in quantitative RT-PCR.

Gene	Forward primer (5' ⟶ 3′)	Reverse primer (5' ⟶ 3′)	Length of product (bp)
GAPDH	CTGGAGAAACCTGCCAAGTATG	GGTGGAAGAATGGGAGTTGCT	138
IL-1*β*	CTCACAGCAGCATCTCGACAAGAG	TCCACGGGCAAGACATAGGTAGC	95
MCP-1	GTCACCAAGCTCAAGAGAGAGA	GAGTGGATGCATTAGCTTCAGA	190
TNF-*α*	CCAGGTTCTCTTCAAGGGACAA	GGTATGAAATGGCAAATCGGCT	80

**Table 2:** Clinical parameters of different groups.

Parameters	C group	DM group	SL group	SM group	SH group
KW/BW (g/100g)	0.28 ± 0.04	0.59 ± 0.04^*∗∗*^	0.47 ± 0.09^##^	0.41 ± 0.05^##^	0.39 ± 0.04^##^
SCr (*μ*mol/L)	24.36 ± 2.70	50.10 ± 4.11^*∗∗*^	43.80 ± 5.89^#^	38.03 ± 5.83^##^	31.49 ± 5.90^##^
BUN (mmol/L)	7.82 ± 0.82	16.05 ± 0.70^*∗∗*^	13.95 ± 1.77^##^	12.05 ± 1.54^##^	10.07 ± 1.35^##^
24 h-UTP (mg/d)	5.76 ± 1.54	42.44 ± 5.93^*∗∗*^	34.79 ± 5.29^##^	29.13 ± 4.96^##^	25.83 ± 5.91^##^
CHO (mmol/L)	2.72 ± 0.29	6.00 ± 0.75^*∗∗*^	5.48 ± 0.63	4.20 ± 0.51^##^	3.54 ± 0.35^##^
TG (mmol/L)	1.09 ± 0.08	3.72 ± 0.56^*∗∗*^	2.70 ± 0.19^##^	2.07 ± 0.18^##^	1.54 ± 0.25^##^

The SL, SM, and SH group rats were dosed by oral gavage once daily for 16 weeks with Hirudo lyophilized powder. The C and DM group rats were administered the same volume of vehicle used to prepare the test medications. Data are presented as means ± SD (*n* = 7 in DM and SL groups, *n* = 8 in the other groups). ^*∗∗*^*P* < 0.01 vs. C group; ^#^*P* < 0.05 and ^##^*P* < 0.01 vs. DM group.

## Data Availability

The data used to support the findings of this study can be obtained from the corresponding author upon request.
